# Allopathic medicine practitioners’ experiences with non-disclosure of traditional medicine use

**DOI:** 10.4102/hsag.v29i0.2381

**Published:** 2024-01-31

**Authors:** Lindiwe Gumede, Pauline B. Nkosi, Maureen N. Sibiya

**Affiliations:** 1 Department of Medical Imaging and Radiation Sciences, Faculty of Health Sciences, University of Johannesburg, Johannesburg, South Africa; 2 Department of Radiography, Faculty of Health Sciences, Durban University of Technology, Durban, South Africa; 3 Faculty of Innovation and Engagement, Mangosuthu University of Technology, Durban, South Africa

**Keywords:** traditional medicine, Allopathic medicine practitioners, non-disclosure, patient treatment outcomes, consultation, stigmatising, belief systems, cultural and ethnic reasons

## Abstract

**Background:**

A pertinent issue impacting patient treatment outcomes is the nondisclosure of traditional medicine (TM) use to Allopathic medicine practitioners (AMPs). For years, TM has been a controversial practice, with patients often using it alongside allopathic medicine without disclosing their use. It is imperitive to learn and understand the experiences of AMPs regarding the disclosure of TM use in Gauteng province to enable them to provide the best possible treatment outcomes for patients who use TM.

**Aim:**

This study aimed to explore the experiences of AMPs regarding non-disclosure of TM use in Gauteng province.

**Setting:**

This study was conducted in four district hospitals where outpatient care and services are rendered in Gauteng Province.

**Methods:**

An interpretive phenomenological analysis (IPA) design was followed. Fourteen purposefully sampled AMPs participated in face-to-face, one-on-one, and semi-structured interviews. Interpretive phenomenological analysis in Atlas.ti was conducted.

**Results:**

Three themes emerged: bedside manner of AMPs; stigmatising TM use; and individual belief systems. The belief of patients’ disclosure hesitancy because of fear of judgment by the AMPs underpinned these themes.

**Conclusion:**

Allopathic medicine practitioners are aware that patients who use TM could feel guilty and stigmatised. They acknowledged that patients use TM because of cultural and ethnic reasons, which should not be disregarded.

**Contribution:**

The study highlighted that patients do not disclose their TM use because of AMPs’ attitudes, stigmatising TM use, and their prejudices against the cultural beliefs of patients. Allopathic medicine practitioners should establish good communication with patients by providing patient-centred communication to facilitate disclosure of TM use.

## Introduction

The focal role in establishing a therapeutic relationship between allopathic medicine practitioners (AMPs) and patients is of effective communication between the two (Pasca 2020). The lack of good communication between AMPs and patients during consultations can discourage disclosure. Therefore, AMPs must have good verbal and nonverbal communication skills to interact with patients. These include active listening, enquiring, communicating, and inspiring others to speak (Jahan & Siddiqui 2019). This is also true for patients who use both traditional medicine (TM) and allopathic medicine (AM) (Gall et al. 2019). When working with various patients, cross-cultural communication, which is part of cultural competence, is frequently required (Jahan & Siddiqui 2019). To prevent communication breakdowns between patients who use TM and AMPs, AMPs must adapt their consultation communication to the patient’s specific requirements. According to a recent study, patients who use both TM and AM may not disclose their use of TM because of poor communication between patients and AMPs, a lack of awareness of the potential interaction between TM and AM, and AMPs incorrectly judging the use of TM (Agarwal 2020). Allopathic medicine practitioners are more likely to overlook serious health issues caused by the negative interaction of TM and AM, as well as a breakdown in the connection between patients (who use both TM and AM) and AMPs, because of a lack of proper communication (Bauer & Guerra 2014). Studies suggest that AMPs must probe patients to encourage disclosure and increase the rate of disclosure of TM use (Thandar et al. 2019; Van der Geest & Hardon 2006).

Over the last decade, there has been little change in the rate of TM use disclosure to AMPs (Foley et al. 2019). However, rates of disclosure of TM use vary according to population demographics and TM classification. A study conducted in Malaysia reveals that patient disclosure was motivated by multiple benefits, including the possibility of receiving advice from AMPs regarding the continuance of TM use or receiving more information about the TM they use (Kelak, Cheah & Safii 2018). The lack of disclosure regarding TM usage holds significance, especially in understanding how AMPs are pivotal guardians in ensuring their patients’ overall health awareness (Foley et al. 2019). According to Johny, Cheah and Razitasham (2017), AMPs tend to be business-oriented and lack human connection with TM patients, which hinders disclosure. Several articles have addressed grounds for non-disclosure of TM use (Agyei-Baffour et al. 2017; Mokhesi & Modjadji 2022; Stubbe 2018). Motivation for non-disclosure may include, but is not limited to, failure of AMPs to inquire about TM use, AMPs reprimanding patients for using TM, and that AMPs have limited knowledge of TM and therefore they would not benefit from disclosing their TM use (Mwaka, Abbo & Kinengyere 2020). Despite documented evidence of barriers to patients’ disclosure of TM use to AMPs, there is a paucity of research examining AMPs’ experiences with non-disclosure of TM use. Therefore, it is important to encourage AMPs to inquire about the patient’s use of TM (Stubbe 2018).

The use of TM is common in South Africa and therefore AMPs need to be knowledgeable to better manage their patients for better outcomes (Zingela, Van Wyk & Pieterse 2019). Patients may believe that their TM use is confidential; therefore, AMPs might not be aware that they are using TM and AM concurrently. To ensure patient safety, a thorough investigation of patients’ disclosure of TM use as well as the reasons for non-disclosure is required (Mokhesi & Modjadji 2022). Currently, existing patient–AMP communication does not allow AMPs to link TM and AM in practice; thus, the development of workable methods to guide the process is required (Wardle, Sibbritt & Adams 2018). These methods could assist AMPs in comprehending patients’ non-disclosure, allowing them to enhance treatment and communication with patients who use TM.

Several studies have examined the impact of involving patients in healthcare decisions. For example, Krist et al. (2017) found that when patients are involved in decision-making, they have a better understanding of their treatment options and are more likely to make informed choices. Similarly, Bombard et al. (2018) argue that involving patients in healthcare decisions can improve communication between patients and healthcare providers, leading to better service delivery and governance. However, despite these findings, there is still a need for further research to explore the implications of non-disclosure or disclosure on treatment outcomes and the provision of care by healthcare providers (Foley et al. 2019).

### Purpose

This study explored the experiences of AMPs regarding the disclosure of TM use in Gauteng province.

## Research methods and design

### Research design and context

A qualitative, interpretive phenomenological research approach guided by hermeneutics was followed to explore the lived experiences of AMPs when consulting with patients who use TM (Rodriguez & Smith 2018; Smith 2004). The chosen design was deemed appropriate for this study because it allowed the researcher to begin comprehending the variety of factors of non-disclosure of TM that can affect AMPs based on their perspective and experience, revealing concealed meaning rather than making inferences. The study was guided by a constructivist paradigm, which took a relativist ontology approach and incorporated various intangible mental concepts that were derived from the contextual and experiential knowledge of the AMPs (Guba & Lincoln 1994). The study was conducted at selected district hospitals in the Gauteng province. Four district hospitals were selected for this study because they promote primary health care and serve as a gateway to specialised care. These hospitals are where most patients with chronic illnesses and potential TM users are managed. This study was inspired by the main researcher’s prior experience as a radiographer, who saw several difficulties faced by AMPs when dealing with patients who might be using TM and not disclosing TM use to them.

### Population and sampling

Population in research is the entire group of individuals with similar characteristics from whom data will be collected (Polit & Beck 2017). The study population were AMPs working in the outpatient departments (OPDs), in the district hospitals in the Gauteng Province. A non-probability, purposive sampling described by Polit and Beck (2017), was adopted to select AMPs in the OPD from multiple sites. The researcher selected participants based on specific demographic characteristics (Table 1) within the population. Using inclusion criteria, the main researcher made inquiries of the AMPs to confirm their eligibility to participate in the study. Seven male and seven female AMPs (N = 25; *n* = 14) who voluntarily consented and had worked at the designated OPDs for at least one year were included in the study. Consequently, only 14 of the 25 AMPs met the inclusion criteria and consented to participate in the study. The participants met the inclusion criteria if they were registered with the Health Professionals Council of South Africa (HPCSA), permanently employed at the institution, and held an undergraduate qualification in medicine with at least one year of experience in general patient administration. All ethnic groups were permitted to participate in the study.

**TABLE 1:** Demographic data of participants.

Characteristics	*n* = 14
**Biological sex** Female (F) Male (M)	7 7
**Age group (years)** > 40 < 40 < 50	3 6 5
**Race** Black Indian	13 1
**Marital Status** Single Married	4 10
**Qualification** Undergraduate Postgraduate	13 1
**Work experience (years)** < 3 > 10 < 15 < 20 < 30	2 6 3 1 2

*Source:* Gumede, L., 2022, ‘Guidelines for disclosure of traditional medicine use to allopathic medicine practitioners by patients who use both traditional medicine and allopathic medicine at selected hospitals in Gauteng, South Africa’, PhD thesis, Durban University of Technology

### Participant recruitment

Participant recruitment is the process by which the researcher solicits the cooperation of prospective participants (Polit & Beck 2017). After obtaining permission from the gatekeeper, the researcher approached AMPs in the OPDs of the selected hospitals to initiate the recruitment process. An information letter explaining the study’s methods, including confidentiality concerns, anonymity processes, and the chance to withdraw at any moment if desired, was given to AMPs. Those AMPs who were interested provided their contact information to the researcher so they could discuss the optimal interview times, dates, and locations. Participants said that meeting outside of business hours would be difficult and that it would be more convenient to conduct the interviews inside hospitals within their respective departments. This decision was acceptable because it enabled the researcher to make insightful field notes across several visits to the chosen research sites and uncomplicated access to the participants.

### Data gathering

All information was gathered following the *Protection of Personal Information (POPI) Act 4 of 2013*, South African Government legislation, and participant contact information was logged against codes created for the study. All participants signed informed consent before data gathering could commence. In a password-protected device, all the information was kept private and confidential.

The main researcher pre-tested the interview guide through a pilot study to maximise the research data and data collection and researcher practice before the actual study. Because of this pilot study, the researcher became acquainted with the research procedure (Majid et al. 2017). The results from the two pilot interviews revealed that the two participants could explain their experiences when consulting with TM patients, so no changes were made to the questions. The data, however, were not included in the main study because the initial objective was to test the interview guide as part of the research design in order to maximise answering of the questions and to determine how the chosen design would affect this (Kara 2022). The pilot also provided sufficient practice in taking field notes, including what, how, and how much to record, and an opportunity to test the audio recorder for the interviews (Shakir & Rahman 2022). The two participants who participated in the pilot study did not participate in the main study (N = 25; *n* = 2).

All interviews were conducted by the main researcher between November 2021 and July 2022. To adhere to coronavirus disease 2019 (COVID-19) control rules, the researchers and participants maintained social distancing, sanitising, and using face masks. One-on-one interviews using a semi-structured interview guide including demographic questions and semi-structured questions were used to facilitate data collection. Probing questions were used based on the participants’ responses. The interviews lasted for 30 min to 45 min each. The interviews were conducted in quiet and private rooms organised by the AMPs within their departments. All the interviews were audio-recorded and none of the participants disagreed to be recorded. Data saturation was reached when the researcher could no longer be presented with new data to add to the information collected from previous interviews (Creswell & Creswell 2023). Data saturation was reached by the12th participant and two more participants were interviewed to confirm the data saturation.

### Data analysis

Before analysis, the entirety of the interview data were transcribed verbatim. To analyse the data, the main researcher used Finlay’s consolidated common strategies and steps in interpretive phenomenological analysis (IPA) (Finlay 2011). This was done to describe the AMPs’ experiences regarding the disclosure of TM in Gauteng province. The methodology was inductive, based on study findings, and acknowledged the preeminent literature (Rodriguez & Smith 2018). The transcriptions were read multiple times to extract and generate meaning. Significant statements were extracted from the interviews in order to discover the meanings of the participants. At this point, the transcripts were uploaded into Atlas.ti which is a computer-assisted qualitative data analysis software, which makes data access and administration simple (Atlas.ti software 2021). A project was created and titled in Atlas.ti as it required opening a Hermeneutic Unit, referred to as the project container within Atlas.ti. The main researcher organised the field notes and the interview transcripts into separate document groups. The researcher highlighted specific passages from the transcripts and utilised Atlas.ti for the abstraction and integration of themes. All these findings delved into the AMPs’ actual interactions with patients who use both TM and AM, and the researchers’ interpretations further shaped them. The researcher recognised that she was a radiographer and acknowledged her own assumptions, views, and opinions regarding the topic to ensure bracketing. The other two researchers reviewed the initial themes and subthemes noting idiosyncratic instances until they were refined into the main themes presented in this manuscript.

### Trustworthiness

The researcher followed Lincoln and Guba’s (1985) defined stages to develop rigour and trustworthiness in this qualitative study, which included credibility, transferability, dependability, and confirmability. The researcher was able to conduct interviews with the participants in their workplace, which allowed for detailed field notes and gave the study credibility. The data provided by participants contains enough descriptions to define the research’s context for transferability. Furthermore, dependability was ensured by pre-testing the interview guide that recorded all interviews, checking all interview transcripts, and taking field notes as needed. To boost confirmability in this study, the researcher used reflexivity by constantly reviewing oneself and the connection with the research. The researcher also kept an audit trail by documenting the techniques for data gathering and analysis.

### Ethical considerations

The Durban University of Technology and Institutional Research Ethics Committee approved the research and provided a clearance number IREC 016/21. Informed consent was obtained from all participants before conducting the interviews. The researcher kept basic ethical principles in mind while conducting the interviews and throughout the study to avoid any deviations from the established protocol, such as inadequate informed consent, subjecting participants to harm (either psychological or physical), and carelessness concerning participant anonymity and confidentiality. Respect, beneficence, and justice were three essential ethical concepts applied to the study (Belmont Report 1978).

## Results

Fourteen (*n* = 14) AMPs (Table 1) working at the selected outpatient departments agreed to participate voluntarily in the face-to-face, one-on-one interviews. Only one (*n* = 1) of the AMPs held a postgraduate qualification in medicine, and 13 (*n* = 13) of the participants had undergraduate qualifications in medicine. Three primary themes and related sub-themes were established upon data analysis reflecting the lived experiences of AMPs regarding non-disclosure of TM use in Gauteng province. The themes and sub-themes that were developed from the data are summarised in Table 2.

**TABLE 2:** Themes and sub-themes.

Themes	Sub-themes
Allopathic bed-side manner	The attitude of AMPs Effective communication of AMP and patient Manner of approach
Stigmatising TM use	Alleviating the stigma Negative AMP responses Witchcraft and TM use
Individual belief systems	Patients trust TM Prejudices against cultural belief

TM, traditional medicine; AMP, allopathic medicine practitioner.

When asked how they dealt with patients who had differing perspectives on medical care, AMPs highlighted many elements that influence their experience during consultation with patients who use TM. The approach and attitude of AMPs towards these patients, which results in barriers to disclosure, is at the heart of the three themes. The AMPs emphasised how important trust was and thought that if a patient disclosed their use of TM and then felt judged, they would not want to go through the same process again.

### Theme 1: APMs’ bedside manner

The AMPs’ bedside manner could promote non-disclosure of TM use by patients. Their attitude when consulting with patients could be the cause of most patients who use TM opting to share only what will be acceptable to the AMPs.

#### Sub-theme 1.1. The attitude of AMPs’

The AMPs described that there is a need to consider their attitude to ensure good practice when interacting with patients who use TM. The AMPs seemed to think that their negative attitude promotes non-disclosure as patients may see this as dismissive when AMPs do not listen to them. The following excerpts supported this sub-theme:

‘… Non-disclosure by traditional medicine patients is due to our attitude as doctors. We do not pay attention to what patients have to say. This is especially true for very traditional work.’ (AMP2/M/68/Physician)

‘… I believe patient disclosure of traditional medicine use is influenced by your bedside manner as a doctor. How do you interact with a patient when he or she enters the room?’ (AMP7/M/45/Physician)

#### Sub-theme 1.2. Effective communication of AMP and patient

The AMPs believe good communication is regarded as an essential part of the interaction between them and patients, as both parties need to be active during a consultation to encourage disclosure of TM use. During the semi-structured interviews, AMPs indicated the following:

‘How doctors communicate with their patients, good communication skills are essential. We must show the patient respect and treat the patient as if he or she were our brother or sister. And be sure to explain your point of view thoroughly. When we undermine traditional medicine’s use due to preconceived notions. We make our patients unable to disclose, and their lives will impede the healing process and cause unnecessary investigating.’ (AMP9/M/51/Physician)

‘… The way the patient should be approached. How the doctor simply asks the patient questions, how the doctor opens up to the patient, and how the patient opens up to you. That, I believe, is how the patient will reveal that they are using traditional medicine … Our attitude toward them as medical professionals lead to non-disclosure, while you have no idea how traditional medicine works.’ (AMP12/F/60/Physician)

#### Sub-theme 1.3. Manner of approach

According to AMPs, the method by which patients who use TM are approached may influence their decision to disclose. This observation shows that AMPs must be courteous and personable when communicating with patients who use TM. As a result, when AMPs approach such patients, they must keep in mind that they may be subjected to various treatment alternatives used by these patients. The AMPs ascertain that:

‘If they [*patients*] have a health care practitioner who asks them or somebody who is less judgmental in their approach of history taking, they might disclose something.’ (AMP/F/12/Physician)

‘All right, a recommendation to healthcare providers. They should be aware of the people they serve. They should be aware of the situation. Traditional methods have been used and taught to people as curative treatments. So, when approaching such a patient, they should be sensitive, understanding that there are various options. Patients should be allowed to exercise their rights to access their treatment of choice.’ (AMP13/M/28/Physician)

### Theme 2: Stigmatising TM use

The second theme that was developed was stigmatising TM use by AMPs during a consultation with patients who use both TM and AM. Allopathic medicine practitioners working with these patients must avoid judging patients because this instils a sense of fear within them. The practice of constantly judging patients who use TM is detrimental and may influence the patient’s trust.

#### Subtheme 2.1: Alleviating the stigma

Some AMPs reported that stigma was one of the factors that discouraged patients from disclosing their use of TM to AMPs. According to their narratives, they believe that incorporating TM into healthcare treatment would help reduce the dread associated with the use of TM. The following quotations back this up:

‘I believe the stigma associated with using traditional medicines will be the first factor in patients’ refusal to disclose their use of traditional medicine.’ (AMP6/F/40/Physician)

‘Technically, if we are to be exposed to say, health care workers, we must spend a week visiting traditional healers to see what they do. The stigma would be reduced as a result, right? We as health care workers need to be exposed to traditional healers or alternatives now that the other stigma has been lifted.’ (AMP1/F/43/Masters in family medicine)

#### Subtheme 2.2: Negative AMP responses

The AMPs indicated that they believe patients might not disclose because they think AMPs would not understand why they chose to use TM. Consequently, patients may fear being held responsible for delayed presentation and the subsequent development of complications. Negative AMP responses are captured from the participants in the following manner:

‘… Patients do not disclose primarily due to responses from healthcare workers. And they are most afraid of being judged. They believe they will face legal consequences for their decision to use traditional medication.’ (AMP3/F/29/Physician)

‘… uhm, I think the most common reason patients do not disclose their use of traditional medicine is uhm … Perhaps they are afraid that health care providers will not understand why they are using it [*referring to TM*].’ (AMP5/M/43/Physician)

‘The reason patients won’t admit to using traditional medicine … Yes, they may fear being blamed for taking those medications. To be held accountable by the doctor or the medical staff. They will be asked why they travel to Sangoma [*THP*]. Why did you decide to use traditional medicine? If they are afraid, they will not reveal.’ (AMP8/M/44/Physician)

#### Subtheme 2.3: Witchcraft and TM use

The association of TM with witchcraft is common; AMPs ascertain that patients are terrified of being judged for making a poor decision that is occasionally supposedly connected to witchcraft. As directly quoted, one of the participants said:

‘Usually, they expect you [*the AMP*] to be angry because you are a doctor. You don’t want them to use anything else but what you’re giving them, and they think you think it’s evil or something. So, won’t disclose, they think you’ll interpret it as if they’re using witchcraft or something shady, and they’ll make you angry when they tell you.’ (AMP11/F/50/Physician)

### Theme 3: Individual belief systems

Individual belief systems and their link to cultural upbringing were verbalised as one of the most challenging points to discuss. The patients’ mindset plays a significant role in how much they may be willing to share, considering how it may be received. This theme was supported by two subthemes: patients trust TM and prejudices against cultural beliefs.

#### Sub-theme 3.1. Patients trust TM

The AMPs acknowledged that most patients that they consult with have a firm trust in TM as South Africans. They alluded that there is not a lot they can do to change the patient’s mindset regarding the use of TM, but they usually provide clarity even though the patients might not agree to discontinue the use of TM at the time. The following quotes illustrate this:

‘I believe traditional medicine is simply a South African way of life, and many South Africans truly believe in traditional medicine, and they will always see a traditional first before coming to see us … I would simply educate them to be cautious of traditional medicine. Because if it is someone’s belief, it is pointless to discourage them.’ (AMP14/M/33/Physician)

‘… I have no objections to that (patients using traditional medicine). It also depends on each person’s belief that it can help them. We are here to assist the patient.’ (AMP4/F/58/Physician)

‘… Traditional medication is trusted and relied on by most patients. You know, if they’ve used it before and it worked, they wouldn’t be shy about disclosing it. They will say, Now, that I’m using it [*the TM*], I know it works, and I can continue to use your medical treatment [*MT*].’ (AMP3/F/29/Physician)

#### Subtheme 3.2: Prejudices against cultural belief

The AMPs expressed that their reaction and response towards patients who use both TM and AM during the consultation was mostly the result of either limited knowledge of how the TM works or no confidence in the TM use. They also highlighted that some patients use TM because of cultural and ethnic reasons, which should not be undermined. In support, the following statements were replayed:

‘In my opinion, the patient must disclose. I encourage it, and once I discover that they are using traditional medicine, I tell them, but I also come from that background, because as Africans, we used to use this traditional medicine. I explain my position, and where I’m coming from, without prejudice, undermining, or implying that traditional medicine practitioners don’t know what they’re doing because, unfortunately, we can’t get rid of them as Africans. Because that is the most important thing, we must accept that traditional medicine will continue to play an important role. It [*TM*] is not something we can eradicate right now. We must not destabilise them [*TM*].’ (AMP9/M/5/Physician)

‘… If the practitioner has prejudices against cultural beliefs and says something about the culture in general and what he believes should be done, the bedside manner or how the practitioner interacts. That patient may feel criticized. Makes the patient believe that, yes, the practitioner has a belief system that contradicts what they believe, or if a practitioner fails to drop to a humane level for a few moments. The practitioner must forget that they are the doctor in the equation and simply interact with the patient as you [*AMP*] would normally.’ (AMP7/M/45/Physician)

## Discussion

This interpretative phenomenological analysis study explored the lived experiences of 14 AMPs regarding non-disclosure of TM use in Gauteng province. In the context of this study, the use of TM without disclosure necessitates proper AMP–patient communication to manage it. This study observed that the AMPs were willing to provide supportive consultations to patients who use TM. The AMPs perceived the disclosure of TM as being guided by the patients’ encounters during consultation. Regarding the experiences of AMPs, the study acknowledged that AMPs must negotiate with patients who use both TM and AM to encourage voluntary disclosure in order to moderate unneeded interactions between the two treatments.

The bedside manner of the AMPs is a significant factor influencing the patient’s decision to disclose or not to disclose the use of TM as highlighted by our work. Prior research in South Africa indicates that patients are convinced that disclosing to AMPs will negatively impact their consultation with the AMPs, resulting in subpar patient care service (Hughes et al. 2012; Puoane et al. 2012). In a recent study about the usage of TM and AM in healthcare facilities, it was discovered that patients lacked adequate engagement and were afraid of AMPs (Mokhesi & Modjadji 2022). Furthermore, bad interactions between patients and AMPs may lead to the patients feeling pitiful, frightened, and demoralised at the same time (Amirudin et al. 2021). Hence, AMPs need to enhance their communication with patients as their negative responses are the main drivers of the fear the patients have for them (Hajj et al. 2020; Amirudin et al. 2021).To improve communication between AMPs and patients about TM use, AMPs must consider the likelihood of their patients using TM and be objective in their interactions with these patients during a consultation (James et al. 2018).

Literature underscores that disclosure hinders AMPs’ negative attitudes, perceived lack of support, unpleasant interactions and behaviours, mistrust, and stigma (James et al. 2018; Kelak et al. 2018). This, in turn, may lead to detrimental effects for AMPs emanating from the bad experience of patients with AMPs (Amirudin et al. 2021; Logiel et al. 2021). Patients who have had bad encounters where AMPs have abused them would not effectively need to return to the same facility for any benefit and would go for TM (Logiel et al. 2021). However, the notion that TM is associated with witchcraft fosters a pessimistic view of TM’s development, fostered by witchcraft suppression laws prohibiting TM practices. (De Lange 2017; Chebii, Muthee & Kiemo 2020; Sifuna 2022).

Traditional medicine is timeless and has formed a significant part of the healthcare system in Africa long before AM was established (Mothibe & Sibanda 2019). There is an acknowledgement of the cultural significance of TM by the older generation (Mokhesi & Modjadji 2022). Therefore, AMPs should consider the individual characteristics of patients, including the individual-level evaluation for integrated patient healthcare (Metin, Karadas & Ozdemir 2019). Notably, the effect of conviction frameworks and particular reasons for TM use among people has progressed in well-being care and spatio-medical writing (Gyasi et al. 2016). It is worth noting that the impact of choosing a health practitioner is a social and devout conviction, following the influence of family or companions (Zingela et al. 2019). Therefore, it could be expected that AMPs that were exposed to a culture of traditional beliefs when growing up would be open to TM practice (Lampiao, Chisaka & Clements 2019).

### Recommendations and future research

The researcher suggests addressing obstacles to improve the therapeutic relationship between patients using TM and AMPs to ensure positive treatment outcomes. Evidence-based interventions should address trust issues and negative responses. Future interventions should identify patients who use TM; improve AMPs’ practices by actively listening; asking questions; and recognising nonverbal cues. Future research could include other healthcare professionals and use a quantitative approach.

### Strengths and limitations

The implementation of interpretive phenomenological analysis (IPA) has yielded contextual insights for the topic’s further investigation. In addition, the discussed interpretations helped shed light on the barriers and facilitators between AMPs and patients who use both TM and AM that were identified in this study. However, the pandemic of COVID-19 hindered the study’s capacity to engage participants for protracted periods. In addition, COVID-19 was cited as one of the reasons why a research site denied access for the research to be conducted. Given that most participants were of black African descent, the results may be biased towards a particular perspective. Despite the numerous limitations encountered during data collection and analysis, the researcher was able to accomplish the purpose of the study.

## Conclusion

There is still a significant amount of research needed in this area to improve the practices used by AMPs during consultations with patients who use both TM and AM. This study provided insight into the opinions of AMPs regarding the nondisclosure of TM use. These findings can contribute to the disclosure and nondisclosure knowledge and offer insights into understanding AMPs’ perceptions about the subject matter. It also provides insight into how AMPs currently perceive patients who use both TM and AM during the consultation and how to avoid increasing non-disclosure of TM use. According to the study, one of the factors that influenced AMPs’ acceptance of TM use by patients who use both TM and AM was tradition and culture. This means that more research is needed to figure out how to improve AMP practices when consulting with a patient who uses both TM and AM and, ultimately, how to make it easier for TM use to be disclosed.
